# An appraisal of lung computer tomography in very early anti-inflammatory treatment of two different ovine ARDS phenotypes

**DOI:** 10.1038/s41598-024-52698-w

**Published:** 2024-01-25

**Authors:** Karin Wildi, Sebastiano Maria Colombo, Daniel McGuire, Carmen Ainola, Silver Heinsar, Noriko Sato, Kei Sato, Keibun Liu, Mahé Bouquet, Emily Wilson, Margaret Passmore, Kieran Hyslop, Samantha Livingstone, Marianna Di Feliciantonio, Wendy Strugnell, Chiara Palmieri, Jacky Suen, Gianluigi Li Bassi, John Fraser

**Affiliations:** 1 Critical Care Research Group, The Prince Charles Hospital, Rode Road, Chermside, Brisbane, QLD 4032 Australia; 2 The University of Queensland, Brisbane, Australia; 3 Cardiovascular Research Institute Basel, University Hospital Basel, University of Basel, Basel, Switzerland; 4 Department of Anaesthesia and Intensive Care Medicine, Fondazione IRCCS Ca’ Granda Ospedale Maggiore Policlinico, Milan, Italy; 5 The Prince Charles Hospital, Chermside, QLD Australia; 6 Department of Intensive Care, North Estonia Medical Centre, Tallinn, Estonia; 7 School of Veterinary Science, The University of Queensland, Gatton, Australia; 8 St Andrews War Memorial Hospital, Intensive Care Unit, Spring Hill, QLD Australia; 9 The Wesley Hospital, Intensive Care Unit, Auchenflower, QLD Australia

**Keywords:** Experimental models of disease, Preclinical research, Respiratory tract diseases

## Abstract

Mortality and morbidity of Acute Respiratory Distress Syndrome (ARDS) are largely unaltered. A possible new approach to treatment of ARDS is offered by the discovery of inflammatory subphenotypes. In an ovine model of ARDS phenotypes, matching key features of the human subphenotypes, we provide an imaging characterization using computer tomography (CT). Nine animals were randomized into (a) OA (oleic acid, hypoinflammatory; n = 5) and (b) OA-LPS (oleic acid and lipopolysaccharides, hyperinflammatory; n = 4). 48 h after ARDS induction and anti-inflammatory treatment, CT scans were performed at high (H) and then low (L) airway pressure. After CT, the animals were euthanized and lung tissue was collected. OA-LPS showed a higher air fraction and OA a higher tissue fraction, resulting in more normally aerated lungs in OA-LPS in contrast to more non-aerated lung in OA. The change in lung and air volume between H and L was more accentuated in OA-LPS, indicating a higher recruitment potential. Strain was higher in OA, indicating a higher level of lung damage, while the amount of lung edema and histological lung injury were largely comparable. Anti-inflammatory treatment might be beneficial in terms of overall ventilated lung portion and recruitment potential, especially in the OA-LPS group.

## Introduction

Since the first description of ARDS in 1967^1^, numerous different research approaches have been trialed for better characterization and understanding of the pathophysiology of the syndrome, aimed to define treatable traits that will ultimately allow a better outcome for patients. Imaging studies have been instrumental in understanding regional lung inhomogeneities in ARDS^2^ and deleterious effects of mechanical ventilation^3,4^, namely ventilation-induced lung injury (VILI)^5^. Consecutively, the recruitment potential of ARDS lungs was assessed with imaging techniques to further understand pathophysiological underpinnings leading to lung damage^6–8^. A landmark study was published in 2006^9^, quantifying the recruitment potential in ARDS and associating high recruitment potential with worse survival rates. Furthermore, the nowadays well-established concept of prone positioning in severe ARDS^10–12^ was studied in translational models using imaging technology^13–15^.

Irrespective of abovementioned findings, ARDS morbidity and mortality remain largely unchanged^16^. A recent discovery might offer an explanation: the heterogeneous population of ARDS patients seems to consist of two subgroups, a hyperinflammatory and a hypoinflammatory subphenotype^17–21^. Post-hoc analyses of clinical trials in ARDS emphasized potentially overlooked benefits of treatments when applied to the hyperinflammatory subphenotype alone^22–24^. Enrichment strategies in general^25^ and in ARDS specifically^26^ will likely guide future studies and therapies towards personalized therapy^27^.

Such enrichment approaches have been tried using imaging technology in ARDS: by differentiating pulmonary and extrapulmonary causes of ARDS^28^ and describing the distribution of aerated lung compartments^29^, Wendel et al. assessed pulmonary phenotypes with imaging and reported 2 subgroups: phenotype 1 was designated by lower lung inhomogeneities, dead space ventilation, and elastance; whereas the hallmarks of phenotype 2 were a higher compliance and recruitment potential but also higher mortality. The study suggests that phenotype 2 might provide at least some overlap with the hyperinflammatory subphenotype, as lower compliance and a higher amount of acidosis have been reported in both. In a recent intervention study^30^ patients were randomized to a personalized ventilation strategy according to their imaging phenotype—focal or non-focal ARDS—but no survival benefit could be demonstrated.

Imaging will be essential to enhance our understanding of the pathophysiology and morphology of ARDS subphenotypes. Therefore, our group developed^31^ and validated^32^ large animal models of ARDS phenotypes, demonstrating that the double-hit model using oleic acid (OA) and lipopolysaccharides (LPS) shares some essential features with the human hyperinflammatory subphenotype. OA alone was pragmatically chosen as the opposite lung injury model with the least systemic inflammation response as compared to OA-LPS. In an intervention study, animals were then treated with anti-inflammatory agents (methylprednisolone or erythromycin) and monitored for up to 48 h. The hyperinflammatory lung injury model treated with methylprednisolone alone was shown an early and consistent improvement in oxygenation^33^. This subanalysis aimed to describe pulmonary volumes (air and tissue) and aeration states in ovine ARDS phenotypes treated with anti-inflammatory agents to test the hypothesis that hyperinflammatory ovine ARDS might benefit from treatment in terms of recruitment potential.

## Materials and methods

Animal studies were conducted at the Queensland University of Technology (QUT) Medical Engineering Facility (MERF) in Brisbane, Australia. The study was assessed and approved by the QUT Office of Research Ethics and Integrity (No 18-606). All experiments were performed in accordance with the Australian Code of Practice for the Care and Use of Animals for Scientific Purposes and the Animal care and Protection Act 2001 (QLD) and complied with the ARRIVE Guidelines^34^.

### Study design

This study was an additional experiment of a study designed to assess the efficacy of anti-inflammatory treatment in two models of ARDS injury in an ovine model (Supplementary Figure S1A)^33^. A lung CT scan was performed in all animals that survived until study end at 48 h if the scanner was available.

### Animal preparation and intra-experimental monitoring/management

All animals were instrumented with a jugular central venous line (CVL) and sheath for Swan Ganz catheter. General anesthesia was induced by using midazolam and propofol intravenously, then the animal was endotracheally intubated. Further instrumentation consisted of femoral arterial line, nasogastric tube, urinary catheter and bilateral pleural drains. Additionally, a surgical tracheostomy was performed in all animals. Lung-protective ventilation was applied as according to the EXPRESS trial^35^. After completing all instrumentation steps, the animal rested for 1 h, then ARDS was induced. Intra-experimental monitoring, management and data collection has been reported in detail before^31,33^.

### ARDS induction and anti-inflammatory treatment

Randomization was performed using a random number generator. Animals randomized to OA received sequential doses of oleic acid (OA, 0.89 g/ml at 25 °C; 01008, Sigma-Aldrich, Australia) intravenously (IV) in subsequent doses of 0.03 ml/kg until a PaO_2_/FiO_2_ ratio (PF) of < 150 mmHg was achieved (= ARDS diagnosis, T0) as assessed by arterial blood gas analysis every 15 min. OA-LPS animals received OA as reported above, after reaching the PF goal, 0.5 µg/kg of lipopolysaccharide (LPS: E. coli O55:B5, Sigma-Aldrich, Australia), dissolved in 50 ml of normal saline was given IV over 1 h.

In animals randomized to treatment with methylprednisolone, 100 mg as a bolus was applied IV at T0, afterwards 2 mg/kg/24 h was administered as a continuous infusion. Erythromycin was applied as 100 mg bolus IV every 6 h after T0. As investigators conducting the clinical experiments were blinded to study treatment, every animal received two boluses of 10 ml at T0 (one containing methylprednisolone 100 mg or normal saline (NS) and one containing erythromycin 100 mg or NS) and a consecutive infusion of 2 ml/h (containing methylprednisolone 2 mg/kg/24 h or NS) as well as a bolus of 10 ml every 6 h (containing erythromycin 100 mg or NS).

### Outcomes

In animals with OA and OA-LPS lung injury, we compared lung volumes and recruitable lung tissue between animals that were treated with anti-inflammatory agents.

### Lung computer tomography at T48h

A Toshiba Acquilion Lightning Computed Tomography Scanner (Canon Medical Care, Japan) was used in this study. Scanner settings were set as previously validated^9^: Acquisition 16 × 1 mm reconstructed to slice thickness 5 mm, interval 5 mm, bed speed 15 mm per second, voltage 135 kV and current non-modulated 240 mA. The sequence of the CT study is shown in Supplementary Figure S1B. Scans were taken at 5 and 45 (arbitrarily defined as total lung capacity) cmH_2_O of airway pressure.

All scans were performed in prone position utilizing consistent scan ranges. First, after administration of 10 mg of vecuronium, pressure-controlled ventilatory mode was used for a recruitment maneuver (RM) set to an inspiratory plateau pressure of 45 cm H_2_O and a PEEP of 15 cm H_2_0 (inspiration to expiration ratio (I:E) 1:1, FiO_2_ 100%, respiratory rate 10 per minute). The maneuver was terminated after 10 breaths and after setting the peak inspiratory pressure to 45 cm H_2_0, the endotracheal tube was clamped during an inspiratory pause and the first whole lung scan performed (high airway pressure: H). After the H scan, the animal was allowed to rest for 10 min, using volume-controlled ventilatory settings as before the RM for respiratory rate, tidal volume and I:E. FiO_2_ left at 100% and PEEP decreased to 5cmH_2_O. After completion of the resting period, the endotracheal tube was clamped during expiratory pause and the second whole lung scan was performed (low airway pressure: L).

### Analysis of cross-sectional lung images

Post-acquisition CT scan analysis was performed with a dedicated software (Maluna 3.17, University Hospital of Göttingen, Göttingen, Germany). Lung CT images were manually outlined including only lung parenchyma and excluding bronchi and large intrapulmonary vessels to measure lung gas content, lung weight and aeration distribution. Further details about CT scan analysis have been provided elsewhere^36–38^. The CT numbers are expressed as Hounsfield Units (HU) which reflect the density of the tissue (volume of gas/volume of tissue), resulting in an estimation of the weight of lung compartments^6^. The distribution of aeration compartments was then assessed as fractions of lung weight and categorized according to HU (gas/tissue content) as not aerated (CT number between + 100 and –100 HU, poorly aerated (CT number between –101 and –500 HU), normally aerated (–501 and –900 HU) and over-aerated (–901 and –1,000 HU). Each slice was then divided into 10 horizontal segments to obtain the ventro-dorsal gradient of density distribution from non-dependent (number 1) to dependent (number 10) to lung regions. Lung tissue weight of each compartment was expressed as a percentage of the total lung weight. We defined recruitment as the variation from non-aerated / poorly aerated region to normally aerated when comparing L and H scans. Dynamic strain was calculated as tidal volume divided by end-expiratory lung volume (EELV, as gas volume at low pressure)^3,39^.

### Measurement of inflammatory cytokines

Arterial blood samples were collected in EDTA blood tubes and processed to plasma, samples were then stored at − 80 °C until batch analysis. Plasma concentration of inflammatory cytokines (e.g. interleukin (IL) -6, -8, -10) were quantified by in-house ELISAs^40^. Positive internal controls were used to ensure that inter- and intra-plate variability was < 10% and to confirm the precision of all the assays.

### Histopathology assessment

Animals were euthanized after the CT scan and lung tissue was taken for histological assessment. Lung tissue was preserved in 10% neutral buffered formalin for 24 h, then embedded in paraffin, sectioned to a 5 µm thickness and stained with hematoxylin&eosin using standard procedures. Slides were examined by an independent, blinded, qualified veterinary pathologist and lung injury score was assessed as recommended by the ATS for experimental ARDS in animal models^41^ (Supplemental Methods and Supplementary Table S1 Online Suppl.). Pulmonary edema was quantified by the wet-to-dry-weight of post-mortem left and right upper and lower lung lobe.

### Statistical analysis

Categorical variables are reported as numbers and percentages and continuous data as mean ± standard deviation (SD). Comparisons between groups were made using Kruskal–Wallis test or Mann–Whitney U test as appropriate. Paired continuous parameters were assessed by a paired sample t-test, while categorical were analyzed by Friedmans 2-way analysis of variance for ranks. All hypothesis testing is two-tailed and p-value of less than 0.05 was considered statistically significant. All statistical analyses were performed with SPSS for Mac 27.0 (SPSS Inc, Chicago, USA).

## Results

### Studied population

Nine female non-pregnant Merino-Dorset crossbreed ewes, aged 1–3 years, with a mean weight of 53 (± 5) kg, were eligible for this analysis. 5 animals were randomized to OA (4 treated with methylprednisolone and 1 treated with erythromycin) and 4 animals to OA-LPS (3 treated with methylprednisolone and 1 with erythromycin) (Supplementary Figure S1A). Baseline characteristics among the two injury models showed no differences (Supplementary Table S2).

### Lung volumes in OA and OA-LPS phenotypes

**Figure 1:**
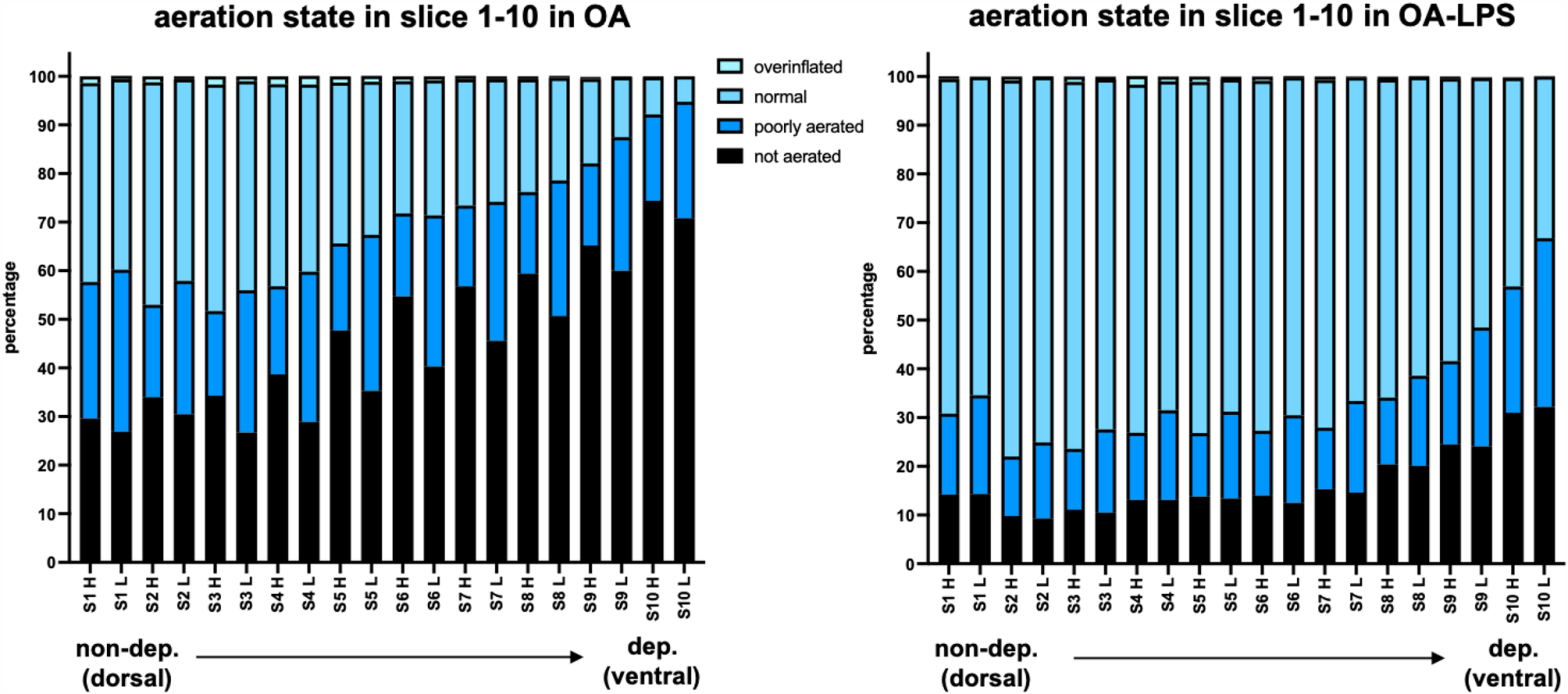
Aeration states in dorso-ventral distribution between high and low airway pressure scans among ARDS phenotypes. H, CT scan performed with high airway pressure; L, CT scan performed with low airway pressure; OA, oleic acid; OA-LPS, oleic acid and lipopolysaccharides. Comparison of the 4 aeration states over slice 1 to 10 for H and L in OA and OA-LPS: OA, (**a**) overinflated L and H *p* < 0.001, (**b**) normal L and H, not aerated H *p* < 0.01, (**c**) not aerated L *p* < 0.05. OA-LPS, (**a**) overinflated L and H, normal L and H, not aerated L and H *p* < 0.01, (**b**) overinflated L < 0.05.

Overall, OA animals showed substantially more non-aerated and poorly aerated lung tissue than OA-LPS, whereas OA-LPS displayed a higher amount of normally aerated lung tissue than OA (*p* < 0.05). The fraction of overinflated lung was low in OA and OA-LPS (Fig. 1, Supplementary Figure S3).

**Figure 2:**
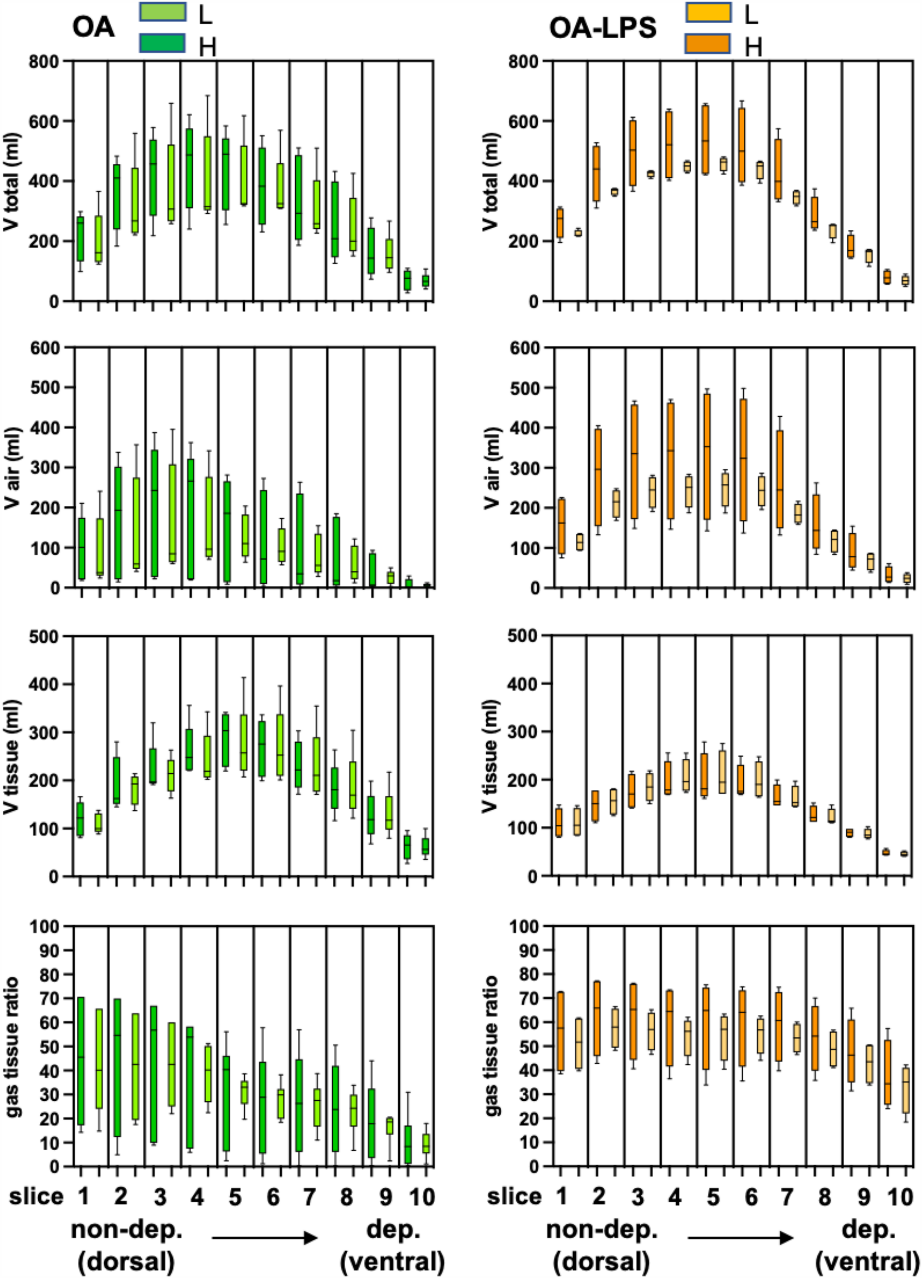
Changes in lung volumes between high and low airway pressure scans among the two ARDS injury types. V, volume; ml, milliliter; H, CT scan performed with high airway pressure; L, CT scan performed with low airway pressure; OA, oleic acid; OA-LPS, oleic acid and lipopolysaccharides.

**Figure 3:**
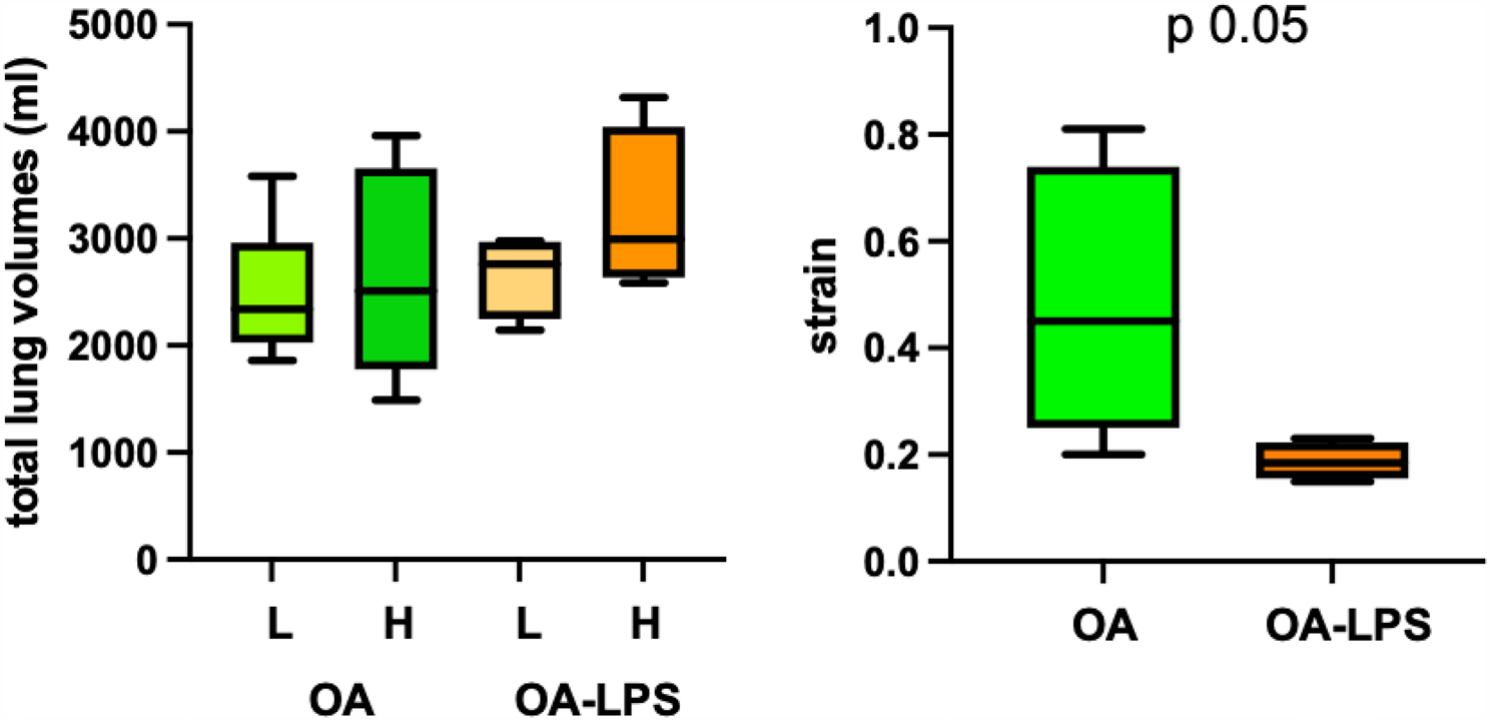
Total lung volumes and strain among different ARDS injury types. H, CT scan performed with high airway pressure; L, CT scan performed with low airway pressure; OA, oleic acid; OA-LPS, oleic acid and lipopolysaccharides.

Total lung volume in OA was 2510 ml (IQR 1778–3657 ml) during H and 2338 ml (IQR 2026–2960 ml) during L, the respective volumes in OA-LPS were 2993 ml (IQR 2634–4043 ml) and 2759 ml (IQR 2249–2696 ml) (Fig. 2). All total lung volumes followed a similar dorso-ventral distribution in OA and OA-LPS, with the highest volumes in slices 2 to 8 (non-dependent to dependent). Distribution of all assessed volumes (total lung volume, air, tissue and gas-tissue ratio) in H and L and in OA and OA-LPS separately, were not the same among slices dorsal to ventral (all *p* < 0.05) (Fig. 2). The total lung volume was comparable among OA and OA-LPS, whereas there was a trend towards higher air fraction in OA-LPS and higher tissue fraction in OA in the non-dependent slices (Fig. 2). Strain was higher in OA (0.45, IQR 0.25–0.74) than in OA-LPS (0.18, IQR 0.16–0.23; *p* 0.05) (Fig. 3).

### Changes in lung volumes according to low and high airway pressure among OA and OA-LPS

The change in total lung volume and air volume between high (H) and low airway pressure (L) per each slice was more accentuated in OA-LPS than OA (p for trend < 0.05), reflecting a higher recruitment potential, whereas the change in tissue volume was comparable among OA and OA-LPS. The change in gas-tissue ratio between L and H was comparable among OA and OA-LPS, but OA-LPS displayed a higher ratio over all slices (Fig. 2; Supplementary Figure S2).

In OA and OA-LPS, there was a higher proportion of normally aerated lung and a lower fraction of poorly aerated lung in H as compared to L (comparison of the 4 aeration states over slice 1 to 10 (non-dependent to dependent) for H and L: p < 0.05). Only in OA animals, the fraction of non-aerated lung was higher in H as compared to L while in OA-LPS it remained largely similar among H and L.

### Clinical and laboratory assessment of animals at 48 h (before CT scan)

Up to 24 h, OA-LPS animals required more vasopressor support, whereas at 48 h (just before the CT scan), they were shown a higher SVRI and lower heart rate with less vasoactive support than OA animals (Table 1). While compliance and EVLWI were comparable among the injury models, driving pressure and dead space ventilation were lower in OA-LPS animals throughout the study. PF ratio remained consistently higher and oxygenation index lower in OA-LPS as compared to OA animals. OA-LPS displayed higher lymphocyte and neutrophil counts than OA (Table 1).

**Table 1:** Characteristics of pulmonary mechanics, hemodynamics and laboratory parameters at 12, 24 and 48 h.

	at 12 h		at 24 h		at 48 h	
	OA (n = 5)	OA-LPS (n = 4)	OA (n = 5)	OA-LPS (n = 4)	OA (n = 5)	OA-LPS (n = 4)
Hemodynamic parameters						
Mean arterial blood pressure (mmHg)	73 (9)	77 (15)	75 (11)	79 (7)	70 (10)	76 (9)
Heart rate (bpm)	73 (18)	97 (15)	96 (19)	82 (7)	95 (30)	66 (7)
Mean pulmonary artery pressure (mmHg)	22 (5)	18 (4)	26 (7)	20 (2)	20 (3)	17 (2)
Cardiac index (L/min/m ^()^)	3.4 (1.1)	3.6 (0.4)	4.7 (0.7)	4.3 (0.7)	5.3 (1.6)	4.0 (0.8)
Systemic vascular resistance index (meena) dyn*s*cm^−5^*m^2^)	1910 (565)	1609 (64)	1252 (337)	1676 (487)	1120 (394)	1484 (262)
Dose of noradrenaline (mcg/kg/min)	0.04 (0.05)	0.15 (0.09)	0.05 (0.07)	0.11 (0.08)	0.15 (0.14)	0.04 (0.05)
Mechanical ventilation						
Minute ventilation (L/min)	8.7 (2.5)	9.8 (1.6)	9.7 (4.6)	9.5 (2.2)	12.1 (3.0)	9.9 (3.7)
Compliance (mL/cmH_2_O)	21 (4)	21 (5)	21 (5)	22 (6)	20 (3)	22 (4)
PEEP (cmH_2_0)	9 (1)	8 (0)	8 (1)	8 (0)	7 (1)	8 (1)
Driving pressure (cm H_2_O)	15 (4)	13 (3)	17 (8)	12 (5)	16 (2)	13 (3)
Extravascular lung water index	40 (12)	48 (6)	43 (13)	41 (7)	45 (2)	42 (6)
Oxygenation index	12 (8)	9 (4)	29 (18)	9 (6)	24 (19)	11 (6)
Dead space ventilation (in ml)	60 (89)	26 (44)	57 (122)	19 (33)	135 (91)	46 (62)
Shunt (%)	29 (14)	22 (6)	18 (5)	25 (5)	15 (4)	24 (7)
Blood gases						
PaO_2_/FiO_2_ ratio	243 (109)	259 (88)	169 (92)	263 (97)	149 (95)	213 (80)
PaCO_2_ (mmHg)	43 (3)	48 (4)	52 (3)	42 (4)	47 (12)	47 (7)
Lactate	2.0 (0.8)	3.1 (0.9)	2.1 (1.8)	2.6 (0.4)	1.9 (0.3)	1.4 (0.5)
Base excess (mmol/L)	1.8 (4.3)	-1.1 (0.8)	-0.2 (6.3)	-1.7 ()1.4	0.4 (2.2)	2.0 (1.8)
Full blood count						
Hemoglobin (g/L)	109 (16)	127 (24)	109 (11)	110 (12)	103 (6)	102 (13)
Platelets (× 10^9/L)	197 (97)	226 (73)	201 (71)	189 (52)	153 (51)	189 (26)
Neutrophil count (× 10^9/L)	3.0 (2.0)	3.6 (1.5)	2.6 (2.8)	4.8 (2.9)	0.2 (2.8)	4.5 (4.8)
Lymphocyte count (× 10^9/L)	1.2 (0.5)	1.7 (0.3)	0.8 (0.4)	1.4 (0.3)	0.7 (0.6)	1.1 (0.6)
Biochemistry						
Potassium (mmol/L)	4.7 (0.4)	5.1 (1.0)	5.2 (0.9)	5.3 (1.0)	4.6 (0.4)	4.8 (0.3)
Creatinine (mmol/L)	0.09 (0.03)	0.09 (0.03)	0.1 (0.07)	0.13 (0.04)	0.09 (0.005)	0.13 (0.05)
Bilirubin (umol/L)	11 (4)	6 (5)	11 (12)	14 (9)	13 (12)	18 (19)
CK (IU/L)	8667 (3889)	8300 (1549)	18,049 (13,363)	19,567 (10,613)	1512 (1317)	10,697 (17,830)
Plasma Cytokines						
IL-6 (pg/ml)	46,531 (17,569)	219,533 (82,887)	40,145 (20,991)	145,060 (54,796)	67,554 (33,134)	64,650 (60,905)
IL-8 (pg/ml)	1171 (934)	490 (235)	2270 (3044)	371 (39)	3135 (1688)	1163 (1479)
IL-10 (pg/ml)	1902 (315)	1889 (499)	2710 (1598)	2093 (752)	2652 (703)	2281 (1580)

All numbers in mean and standard deviation (SD).

PEEP, positive end-expiratory pressure; PaCO_2_ (mmHg), arterial carbondioxide partial pressure; CK, creatine kinase; IL, interleukin; OA, oleic acid; OA-LPS, oleic acid and lipopolysaccharides.

### Histopathology assessment

**Figure 4:**
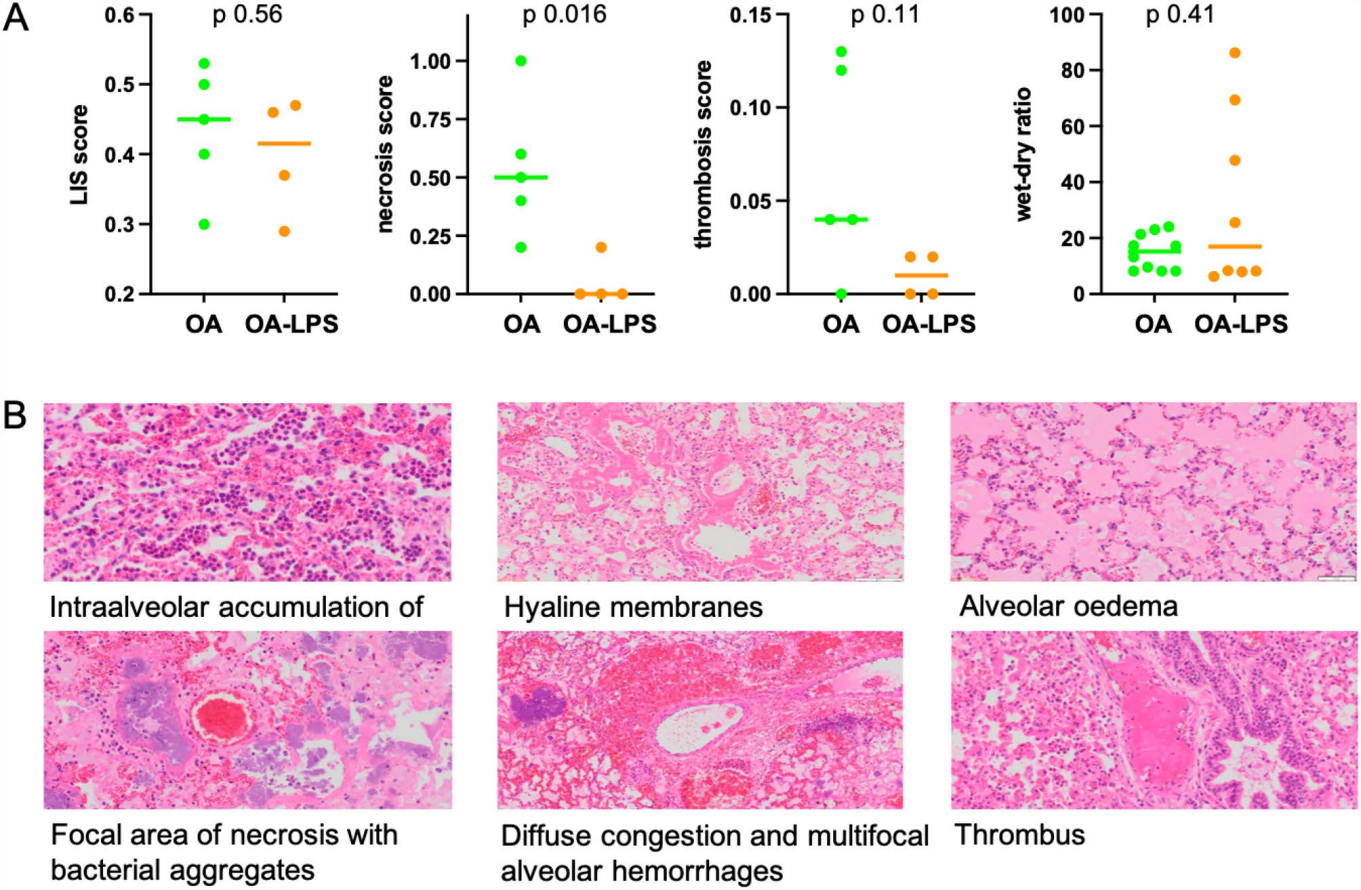
(**A**) detailed histopathological assessment and (**B**) representative examples of findings. LIS, lung injury score; OA, oleic acid; OA-LPS, oleic acid and lipopolysaccharides.

The mean LIS was 0.44 (± 0.09) for OA and 0.40 (± 0.08) for OA-LPS (p = ns). Necrosis score was 0.54 (± 0.3) in OA and 0.05 (± 0.1) in OA-LPS (p 0.016) (Fig. 4A). More bacterial colonies were found in OA (3/5 animals) than in OA-LPS (0 animals), and the presence of alveolar hemorrhage was described in 3/5 animals in OA and 1/4 in OA-LPS (Fig. 4B). Wet-dry ratio was comparable among OA (mean 9.5 ± 2.2) and OA-LPS (mean 7.7 ± 0.9) (p = ns).

### Levels of inflammatory cytokines

Levels of IL-6 were significantly higher in OA-LPS at 12 and 24 h as compared to OA, but assimilating towards study end. Levels of IL-8 and IL-10 were slightly higher in OA than OA-LPS animals throughout the observation period (Table 1).

## Discussion

This is an innovative preclinical imaging study of 2 different ovine ARDS phenotypes treated with anti-inflammatory agents. This study represents the first animal report assessing CT characterization of different ARDS phenotypes, reporting on six clinically relevant findings.

*First,* the highest lung volumes were seen in non-dependent lung areas in both injury types, following a gravitational distribution. *Second,* the total lung volume was comparable among lung injury types, while there was a higher air fraction in OA-LPS and a higher tissue fraction in OA. Thus, there was an increased fraction of normally aerated lungs in OA-LPS in contrast to a higher fraction of non-aerated lung in OA. *Third,* the change in lung and air volume between H and L was more accentuated in OA-LPS than OA, indicating a higher recruitment potential. In contrast, the change in tissue volume from H to L was comparable among OA and OA-LPS. *Fourth,* in response to recruitment maneuvers, both phenotypes showed more normally aerated and less poorly aerated lung in H as compared to L. However, in OA alone, the fraction of non-aerated lung increased at H when compared to L while it remained similar in OA-LPS. *Fifth,* strain was higher in OA, indicating a higher level of lung damage as compared to OA-LPS. *Sixth*, the amount of lung edema and histological lung injury were comparable among OA and OA-LPS, although there was more necrosis and alveolar hemorrhage in OA, indicating a severe lung damage. There was a trend towards higher levels of plasma cytokines (IL-8 and -10), worse hemodynamic and pulmonary mechanics parameters in OA, indicating that these animals were sicker at 48 h into the study.

OA-LPS animals demonstrated more recruitment potential than OA, while generally displaying more normally aerated lung tissue. These findings are in agreement with the physiological results obtained by blood gas analysis and mechanical ventilation data: driving pressure, dead space ventilation and parameters of oxygenation (PF ratio, oxygenation index) displayed a trend towards improved values in OA-LPS animals as compared to OA. While these results are also partly influenced by other variables (i.e., sicker animals in OA with worse hemodynamic conditions), histopathology evidenced differences between lung injury types. OA animals were shown more tissue necrosis, alveolar hemorrhage and bacterial colonies than OA-LPS, potentially indicating a beneficial effect of corticosteroids in the double-hit model. Corticosteroids express their anti-inflammatory potential through many different signaling pathways^42^, affecting genomic and non-genomic levels in their action. The nuclear factor-kappa B pathway (NF-kB) in particular, one of the known main drivers of local and systemic inflammation in ARDS^43,44^, is inhibited by corticosteroids. LPS but not OA activates NF-kB^45,46^, offering a partial explanation why treated OA-LPS animals displayed more benefit in terms of pulmonary inflammation and therefore associated recruitable lung tissue. The throughout higher neutrophil count in OA-LPS is likely a direct effect of endotoxin application, it is possible that this reduced the formation of bacterial settlement in the lungs.

OA displayed higher levels of strain. Lung strain represents pulmonary tissue deformation defined as the ratio between tidal volume and End-Expiratory Lung Volume (Vt/EELV)^3^. A threshold limit in global pulmonary strain has been identified in preventing lung edema and related VILI, thus reducing systemic inflammation and multiorgan dysfunction^3^. Retamal et al. correlated regional volumetric lung strain with regional inflammation in a 2-hit ARDS model in pigs, evaluating CT and PET imaging simultaneously. The study reported evidence that some lung regions may be exposed to even higher values of regional strain than the global estimated one^47^. Lung edema caused by higher strain led to more non- or poorly aerated tissue. However, our results showed no clear association between a higher amount of pulmonary edema (EVLWI, WD ratio) and strain in both phenotypes.

By applying latent class analysis to parameters of gas exchange, respiratory mechanics and CT-derived lung volumes in 238 patients, Wendel et al.^48^ identified two pulmonary phenotypes: phenotype 1 (non-recruitable) and 2 (recruitable). Phenotype 2 was characterized by more ventilated tissue, higher compliance, and PF ratio after recruitment maneuvers. As inflammatory and laboratory parameters were not recorded, it can only be speculated to what extent these phenotypes overlap with the hyper- and hypoinflammatory subphenotypes as described previously^17–21^. The lower compliance, more severe acidosis, and higher ICU mortality as seen in the recruitable phenotype match the hyperinflammatory subphenotype as described by Calfee et al., the distribution of required vasopressor dosage, PF ratio, and ICU scores, however, do not match. Although there is no imaging available in the datasets that identified hyper- and hypoinflammatory subphenotype^17–21^, the response to PEEP as seen in the hyperinflammatory subphenotype^17^ indicates at least some overlap with the recruitable phenotype.

Our ovine model of hyperinflammatory ARDS has previously shown a substantial overlap with the human hyperinflammatory subphenotype^31^. We hypothesize that treatment with anti-inflammatory agents, improved inflammatory response, hemodynamic parameters and metabolic condition in ovine OA-LPS, and resulted in more normally aerated and recruitable lung tissue overall as well as less strain. The next step in translation into clinical practice ought to be personalized phenotyping of human ARDS subphenotypes incorporating omics data and imaging techniques to build a thoroughly comprehensive understanding. Urgently needed is (a) an easily applicable biomarker to identify subphenotypes at bedside and (b) imaging data that informs about recruitment potential and regional lung inhomogeneities. This could allow clinicians treatment allocation of specific ARDS subphenotypes and assessment of recruitment potential for application of safer ventilation strategies for improvement of gas exchange without adding VILI.

Important strengths of this study incorporate the use of an established model of ovine ARDS subphenotypes and a controlled setting with allocation concealment and blinding to treatment.

We report several limitations: *First,* as this was a concomitant study, the small number of animals per group available for assessment, results in an exploratory and hypothesis-generating study that will need confirmation in a larger setting. *Second,* as only animals with survival up to 48 h were eligible for CT scan, a selection bias is possible. *Third,* this study did not include imaging of control animals. Therefore, the possible benefit of anti-inflammatory treatment overall cannot be confirmed in scans from untreated ARDS animals. Fourth, although the used CT analysis method has already been validated in establishing aeration compartiments of the lung in both human and animal studies, this technique has several intrinsic limitations based on assumptions on voxel analysis^7^. *Fifth*, there is no imaging available at baseline, therefore we can only hypothesize that observed differences are the result of a higher responsiveness to anti-inflammatory treatment rather than a consequence of the used double-hit model. *Sixth*, the shape of the chest wall in sheep is different than in humans with consecutively different elastance properties.

## Conclusion

Anti-inflammatory treatment might be beneficial in terms of overall ventilated lung portion and recruitment potential, especially in the OA-LPS group.

### Supplementary Information

Supplementary Information.

## Data Availability

The dataset generated and analysed during the current study are not publicly available due to being part of a PhD project but are available from the corresponding author on reasonable request.

## Abbreviations

ARDS: Acute respiratory distress syndrome
ATS: American thoracic society
CT: Computer tomography
EELV: End-expiratory lung volume
ELISA: Enzyme-linked immunoassay
FiO_2_: Inspired oxygen fraction
H: CT scan performed with high airway pressure
HU: Hounsfield unit
HR: Heart rate
IL: Interleukin
IQR: Interquartile range
IV: Intravenously
L: CT scan performed with low airway pressure
LIS: Lung injury score
LPS: Lipopolysaccharide
MAP: Mean arterial pressure
mPAP: Mean pulmonary artery pressure
NF-kB: Nuclear factor-kappa B pathway
NS: Normal saline
OA: Oleic acid
OA-ctr: OA control
OA-pred: OA treated with methylprednisolone
OA-ery: OA treated with erythromycin
OA-LPS: Oleic acid and lipopolysaccharides
OA-LPS-ctr: OA-LPS control
OA-LPS-pred: OA-LPS treated with methylprednisolone
OA-LPS-ery: OA-LPS treated with erythromycin
Pdriv: Driving pressure
PF ratio: PaO_2_/FiO_2_ ratio
PEEP: Positive end-expiratory pressure
QUT: Queensland University of technology
RM: Recruitment maneuver
SD: Standard deviation
VILI: Ventilation-induced lung injury
