# Supplementary material for: An appraisal of lung computer tomography in very early anti-inflammatory treatment of two different ovine ARDS phenotypes
Source: Sci Rep. 2024 Jan 25;14:2162. doi: 10.1038/s41598-024-52698-w (PMC10810785; doi:10.1038/s41598-024-52698-w)
Supplement: Supplementary file 1 — Supplementary Information. [file 41598_2024_52698_MOESM1_ESM.docx]

**ONLINE SUPPLEMENT**

**Supplemental Methods**

1. **ARRIVE guidelines: checklist**
2. **Study design (A) and time line of experiment (B): Supplementary Figure S1**
3. **Composition of LIS score and Supplementary Table S1**

**Supplemental Results**

**4) Baseline characteristics: Supplementary Table S2**

**5) Mean volume change from high to low airway pressure scans among OA and OA-LPS: Supplementary Figure S2**

**6) Mean aeration state for high and low airway pressure scan among OA and OA-LPS: Supplementary Figure S3**

**Supplemental references**

**Supplemental methods**

**1) ARRIVE guidelines: checklist** (1)

|  | | ITEM | RECOMMENDATION | Section/ Paragraph |
| --- | --- | --- | --- | --- |
| 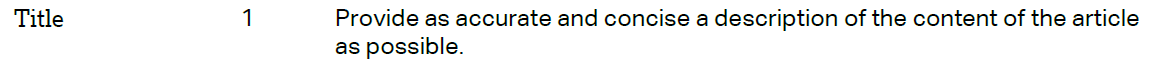 | | | Page 1 |  |
| 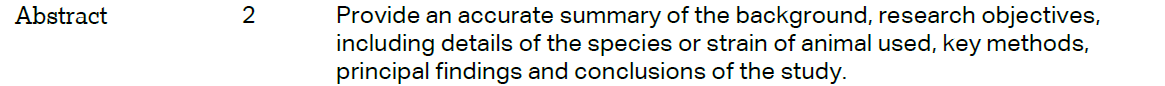 | | | Page 3 |  |
| INTRODUCTION | | |  |  |
| 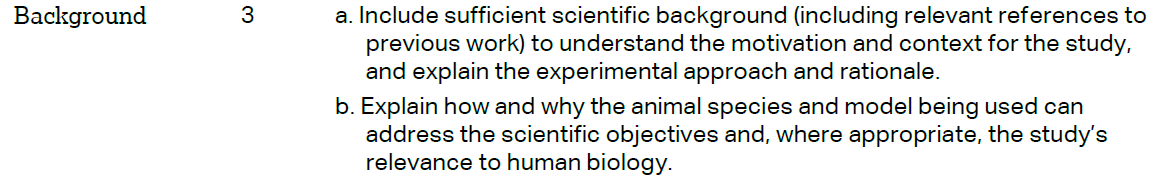 | | | Page 5 |  |
| 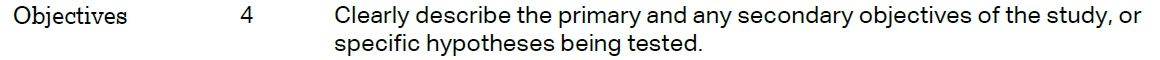 | | | Page 6 |  |
| METHODS | | |  |  |
| 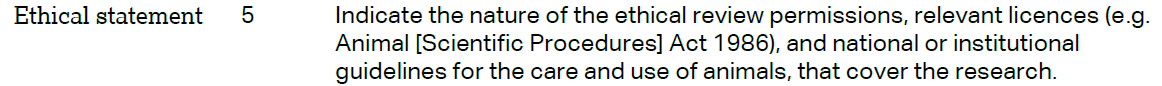 | | | Page 7 and |  |
| 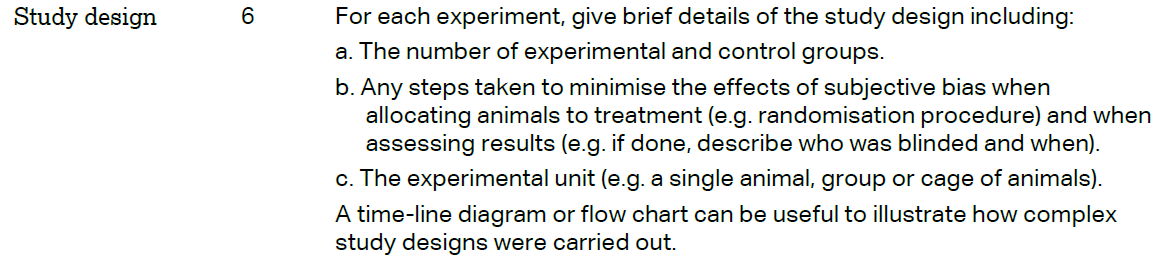 | | | Page 11  Page 7  Page 7  Figure S1 |  |
| 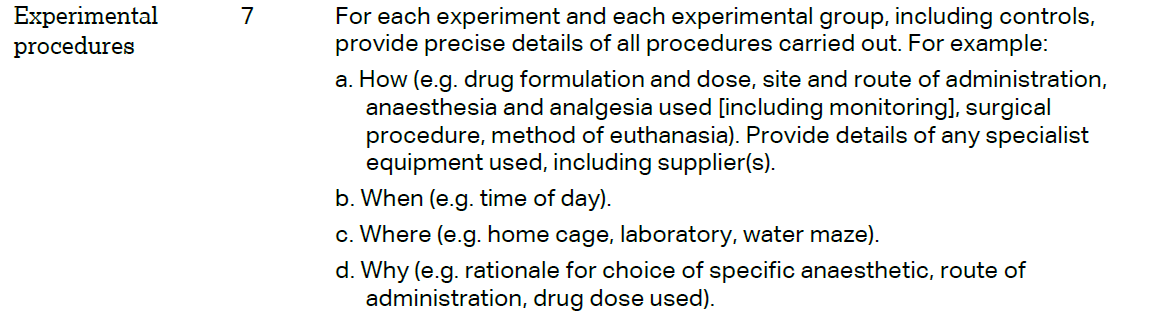 | | | Page 7-8  Page 6 |  |
| 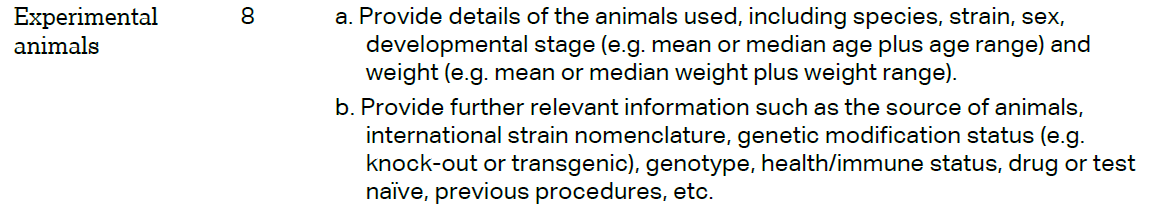 | | | Page 11  NA |  |
| 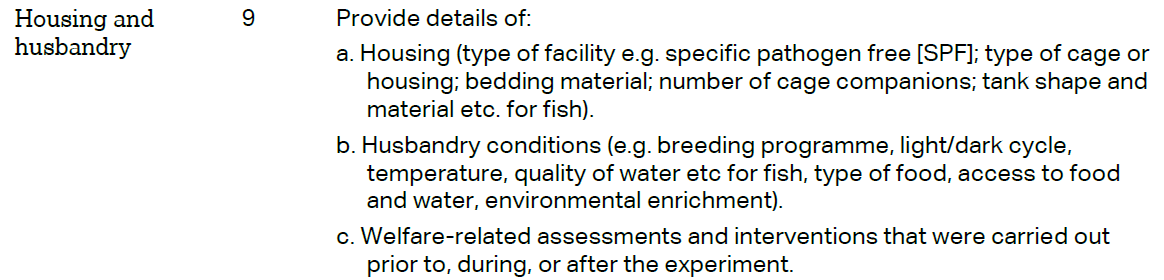 | | |  |  |
| 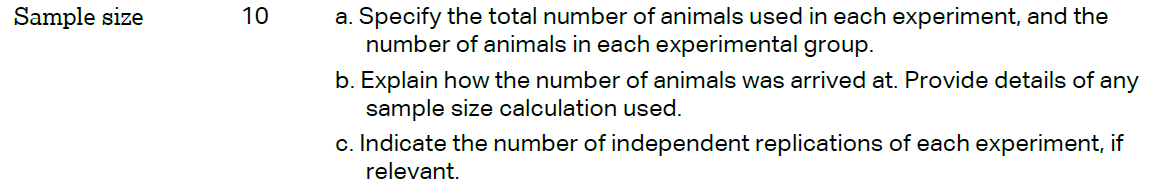 | | | Page 11  Page 11  NA |  |
| 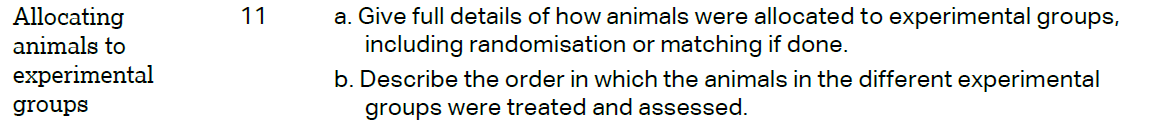 | | | Page 11 |  |
| 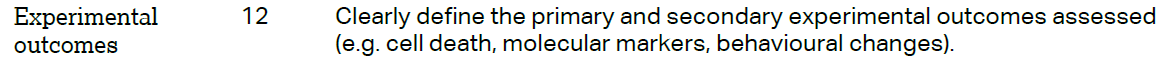 | | | Page 8 |  |
| 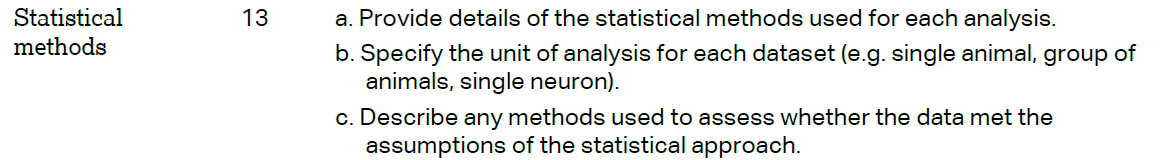 | | | Page 9-10  Page 10 |  |
| RESULTS | | |  |  |
| 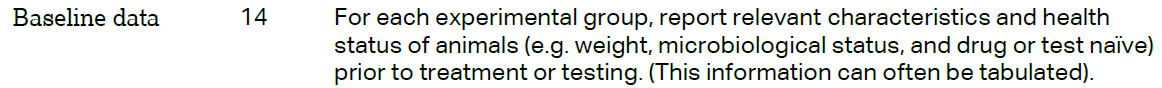 | | | Table S2 |  |
| 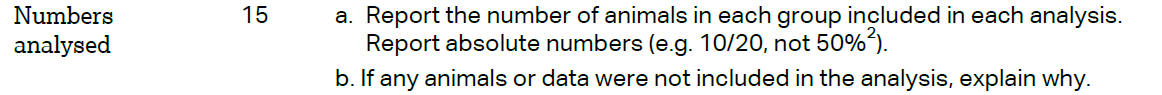 | | | Page 11 |  |
| 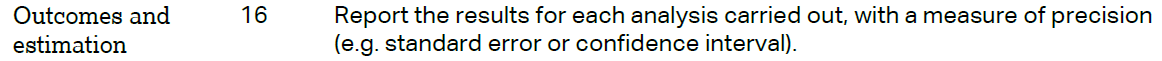 | | | Page 11-12 |  |
| 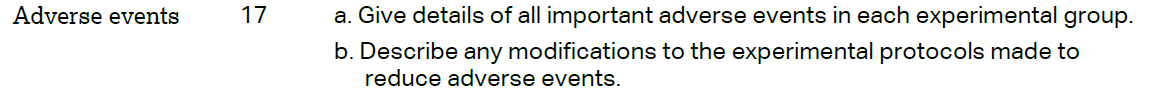 | | | NA |  |
| DISCUSSION | | |  |  |
| 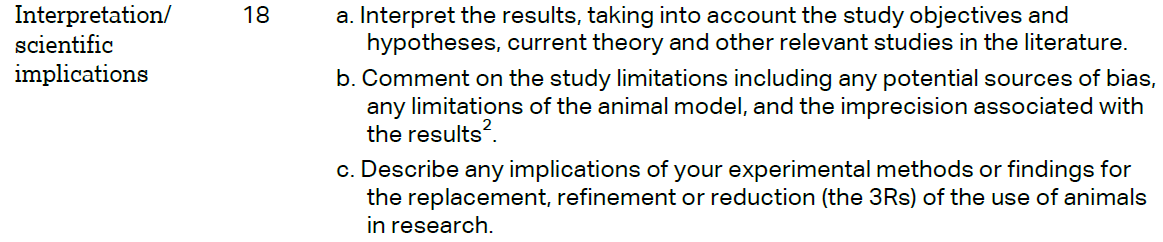 | | | Page 13  Page 15  Page 14 |  |
| 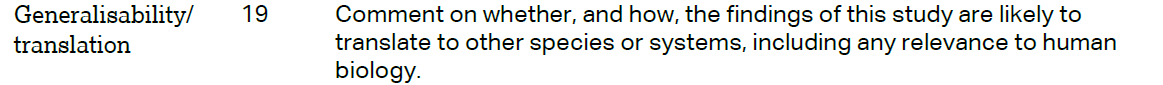 | | | Page 14 |  |
| 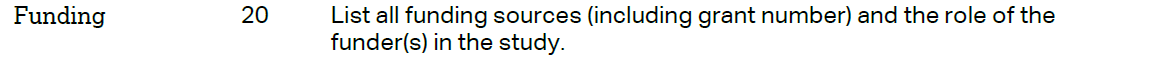 | | | Page 16 |  |

1. **Study design (A) and time line of experiment (B): Supplementary Figure S1**

**
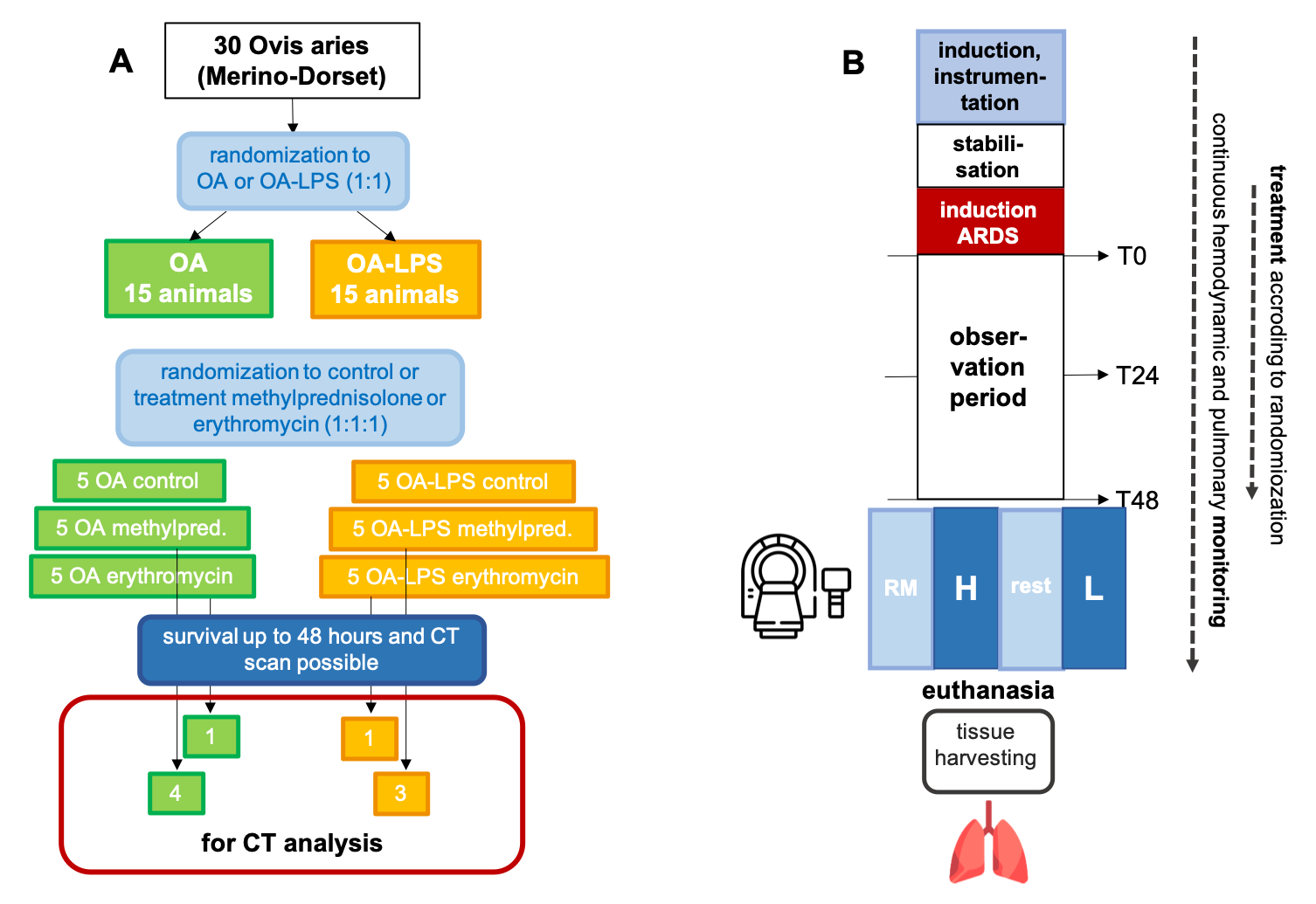
**

**Abbreviations:** ARDS = Acute Respiratory Distress Syndrome; OA = oleic acid; OA-LPS = oleic acid and lipopolysaccharides; CT = computer tomography; H = CT scan performed with high airway pressure; L = CT scan performed with low airway pressure; RM = recruitment maneuver

1. **Composition of LIS score**

Samples were considered adequate for the histological assessment (good quality of the staining, good preservation of the cell morphology). After an initial screening of the lesions, only 2 sections/site were considered (e.g., LLL1 and LLL2). The lung injury score (LIS) system by the American Thoracic Society Consensus Committee (2) scored neutrophils in the alveolar space (A), neutrophils in the interstitial space (B), hyaline membranes (C), proteinaceous debris filling the airspace (D) and alveolar septal thickening (E). Every item was given a score between 0 to 2. The score was calculated by: ((20 x A) + (14 x B) + (7 x C) + (7 x D) + (2 x E))/number of fields x 100, leading to a score between zero (no injury) and one (severe lung injury). Twenty random high-power fields (400x total magnification) were scored per section, and the LIS was calculated per animal (mean ± SD).

For the scoring, at least 50% of each field were occupied by lung alveoli: fields consisting predominantly of the lumen of large airways or vessels were rejected. Septal thickness was not evaluated in alveolar septa directly adjacent to a blood vessel or airway (normally thickened by the collagen present in the peribroncho-vascular bundle).

In addition, the following parameters were evaluated:

- Number of thrombi within blood vessels (score/field: 0 = none; 1 = 1 thrombus; 2 = > 1 thrombus)
- Percentage of section effaced by necrosis (score/field: 0 = none or less than 10%; 1 = 10-50%; 2 = >50%).

**Table S1:** Lung Injury Scoring (LIS) System


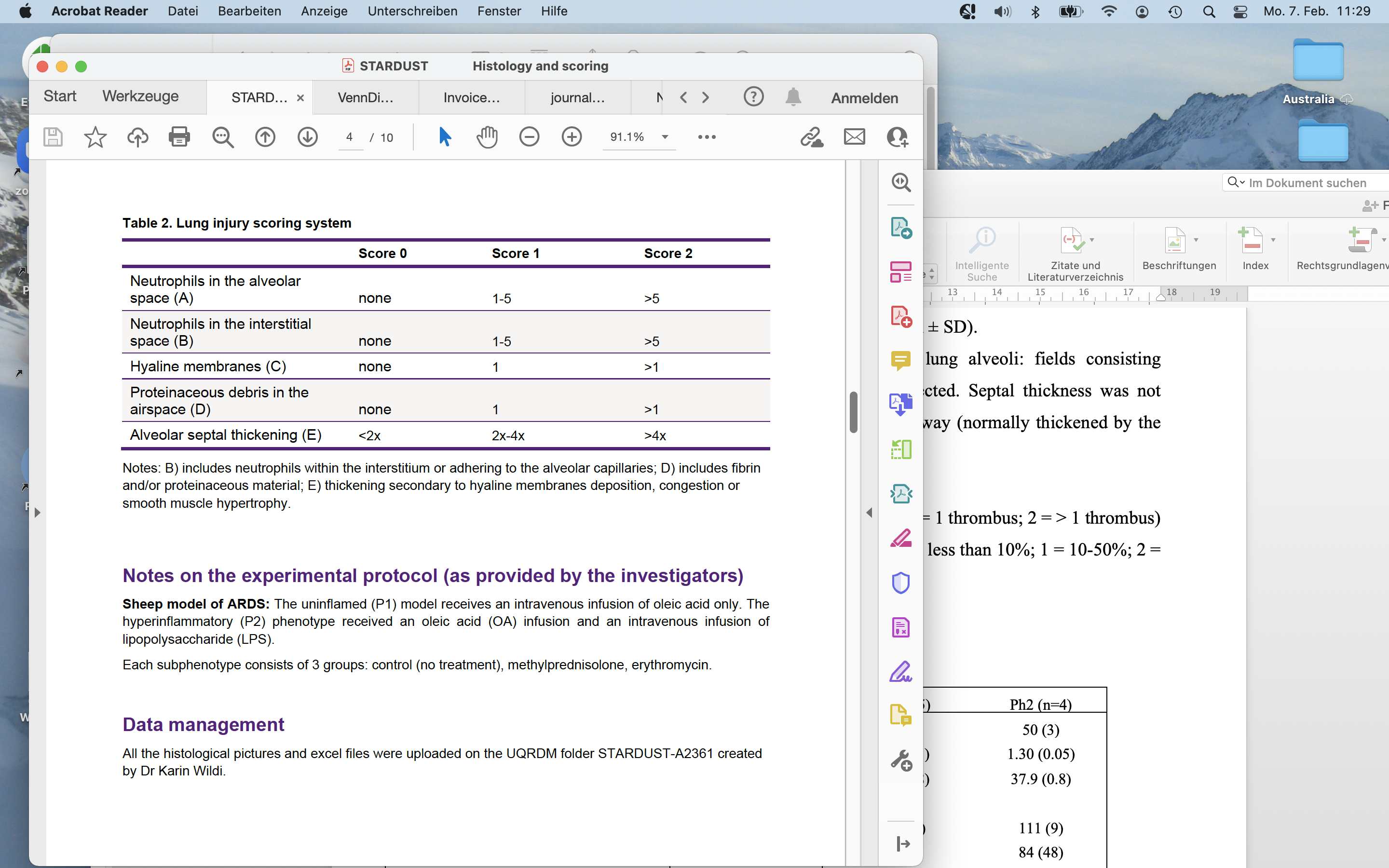


Notes: B) includes neutrophils within the interstitium or adhering to the alveolar capillaries; D) includes fibrin and/or proteinaceous material; E) thickening secondary to hyaline membranes deposition, congestion or smooth muscle hypertrophy

1. **Baseline characteristics: Supplementary Table S2**

|  | all (n=9) | OA (n=5) | OA-LPS (n=4) |
| --- | --- | --- | --- |
| Weight (kg) | 53 (6) | 56 (6) | 50 (3) |
| Body surface area (per m^2^) | 1.34 (0.09) | 1.39 (0.1) | 1.30 (0.05) |
| Temperature (°C) | 38.0 (0.8) | 38.0 (0.8) | 37.9 (0.8) |
| Hemodynamic parameters |  |  |  |
| Mean arterial blood pressure (mmHg) | 113 (16) | 115 (22) | 111 (9) |
| Heart rate (bpm) | 83 (44) | 82 (48) | 84 (48) |
| Mean pulmonary artery pressure (mmHg) | 15 (5) | 18 (4) | 11 (2) |
| Cardiac index | 4.6 (1.8) | 4.6 (2.0) | 4.6 (1.7) |
| Systemic vascular resistance index | 1989 (661) | 2273 (690) | 1635 (479) |
| Mixed-venous oxygenation (%) | 74 (12) | 69 (15) | 80 (5) |
| Mechanical ventilation |  |  |  |
| Minute ventilation (L/min) | 6.4 (0.8) | 6.6 (0.9) | 6.2 (0.5) |
| Compliance (mL/cmH_2_O) | 42.2 (6.7) | 41.7 (6.7) | 42.8 (7.8) |
| PEEP (cmH_2_0) | 5 (0) | 5 (0) | 5 (0) |
| Plateau pressure (cm H_2_O) | 14.5 (1.5) | 15.1 (1.1) | 13.8 (1.7) |
| Extravascular lung water index | 28 (9) | 27 (11) | 29 (6) |
| Blood gases |  |  |  |
| PaO_2_/FiO_2_ ratio | 523 (46) | 507 (50) | 544 (34) |
| PaCO_2_ (mmHg) | 38.4 (4.3) | 37.5 (4.9) | 39.5 (3.7) |
| Lactate | 0.8 (0.4) | 1.0 (0.4) | 0.6 (0.2) |
| Base excess (mmol/L) | 1.7 (2.1) | 0.9 (2.1) | 2.6 (2.0) |
| Bicarbonate (mmol/L) | 25.5 (2.1) | 24.7 (1.7) | 26.5 (1.5) |
| Full blood count |  |  |  |
| Hemoglobin (g/L) | 115 (9) | 111 (9.0) | 120 (7) |
| Platelets (x10^9/L) | 359 (125) | 352 (156) | 367 (96) |
| Neutrophil count (x10^9/L) | 2.1 (0.9) | 2.0 (0.6) | 2.3 (1.2) |
| Lymphocyte count (x10^9/L) | 3.7 (0.6) | 3.5 (0.7) | 3.9 (0.4) |
| Biochemistry |  |  |  |
| Sodium (mmol/L) | 143 (2) | 143 (2) | 143 (3) |
| Potassium (mmol/L) | 4.3 (0.4) | 4.3 (0.5) | 4.4 (0.3) |
| Creatinine (mmol/L) | 0.08 (0.1) | 0.08 (0.01) | 0.07 (0.01) |
| Urea (mmol/L) | 7.2 (1.5) | 7.7 (1.7) | 6.5 (1.0) |
| Albumin (g/L) | 34 (3) | 32 (4) | 35 (3) |
| Bilirubin (umol/L) | 3 (1) | 2 (1) | 3 (1) |
| ASAT (IU/L) | 87 (26) | 75 (25) | 103 (19) |
| ALP (IU/L) | 136 (59) | 136 (69) | 136 (54) |
| gGT (IU/L) | 45 (7) | 43 (6) | 49 (8) |
| GLDH (IU/L) | 14 (10) | 9 (4) | 21 (13) |
| CK (IU/L) | 139 (51) | 123 (47) | 160 (55) |

All data presented as mean and SD

**Abbreviations:** OA = oleic acid; OA-LPS = oleic acid and lipopolysaccharides; PEEP = positive end-expiratory pressure; PaCO_2_ (mmHg) = arterial carbondioxide partial pressure; ASAT = aspartate transaminase; ALP = Alkaline Phosphatase; gGT = gamma-glutamyl transferase, GLDH = glutamate dehydrogenase; CK = creatine kinase

1. **Mean volume change from high to low airway pressure scans among OA and OA-LPS: Supplementary Figure S2**

**
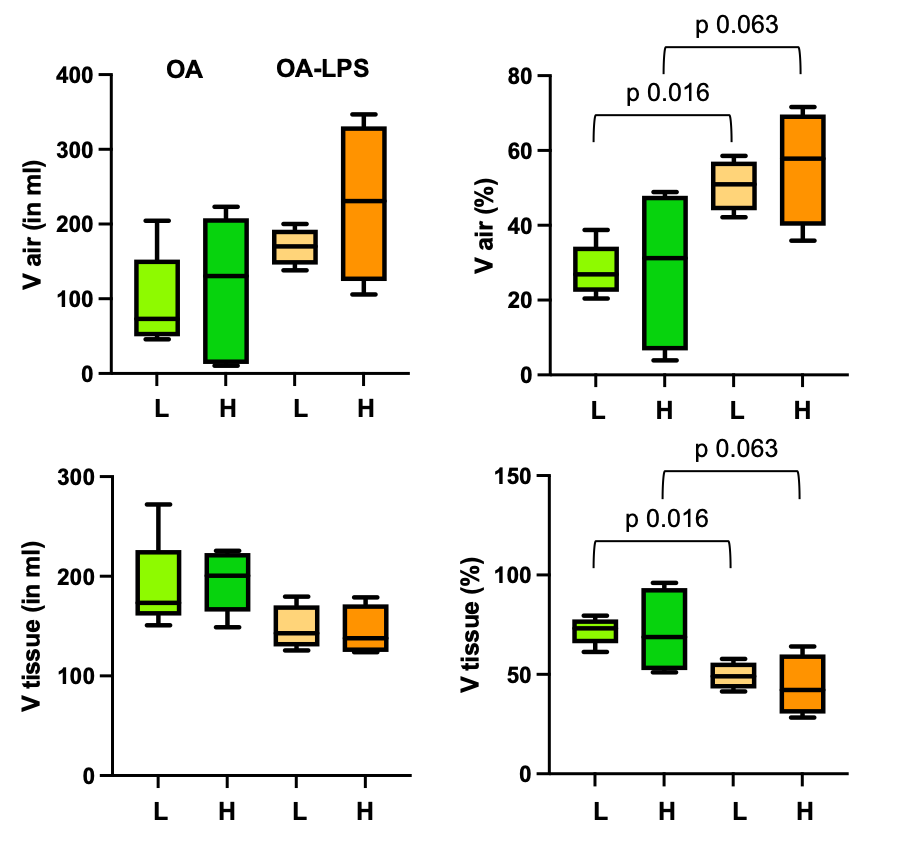
**

**Abbreviations:** OA = oleic acid; OA-LPS = oleic acid and lipopolysaccharides; V = volume; H = high airway pressure scan; L = low airway pressure scan

1. **Mean aeration state for high and low airway pressure scan among Oa and OA-LPS: Supplementary Figure S3**

**
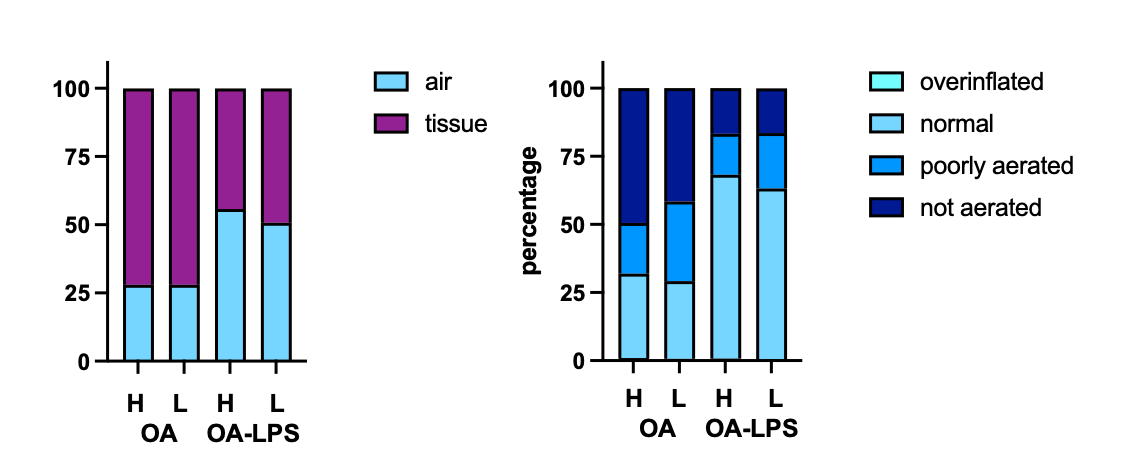
**

**Abbreviations:** OA = oleic acid; OA-LPS = oleic acid and lipopolysaccharides; H = high airway pressure scan; L = low airway pressure scan

**Supplemental references**

1. Percie du Sert N, Ahluwalia A, Alam S, Avey MT, Baker M, Browne WJ, Clark A, Cuthill IC, Dirnagl U, Emerson M, Garner P, Holgate ST, Howells DW, Hurst V, Karp NA, Lazic SE, Lidster K, MacCallum CJ, Macleod M, Pearl EJ, Petersen OH, Rawle F, Reynolds P, Rooney K, Sena ES, Silberberg SD, Steckler T, Würbel H. Reporting animal research: Explanation and elaboration for the ARRIVE guidelines 2.0. In: Boutron I, editor. *PLOS Biol* 2020;18:e3000411.

2. Matute-Bello G, Downey G, Moore BB, Groshong SD, Matthay MA, Slutsky AS, Kuebler WM. An Official American Thoracic Society Workshop Report: Features and Measurements of Experimental Acute Lung Injury in Animals. *Am J Resp Cell Mol Biol* 2011;44:725–38.
